# A randomized controlled trial comparing Circle of Security Intervention and treatment as usual as interventions to increase attachment security in infants of mentally ill mothers: Study Protocol

**DOI:** 10.1186/1471-244X-14-24

**Published:** 2014-01-30

**Authors:** Brigitte Ramsauer, Annett Lotzin, Christine Mühlhan, Georg Romer, Tobias Nolte, Peter Fonagy, Bert Powell

**Affiliations:** 1 Department of Child and Adolescent Psychiatry, Psychotherapy and Psychosomatics, University Medical Center (UKE) Hamburg-Eppendorf, Martinistrasse 52, 20246 Hamburg, Germany; 2 Department of Child and Adolescent Psychiatry, Psychotherapy and Psychosomatics, University Hospital Münster, Schmeddingstrasse 50, 48149 Münster, Germany; 3 Anna Freud Centre, 12 Maresfield Gardens, London NW3 5SU, UK; 4 Educational and Health Psychology Department of Clinical, Educational and Health Psychology, University College London, Chandler House, 1-19 Torrington Place, London WC1E 7HB, UK; 5 Marycliff Institute, 35 W Main Ave, Spokane, WA, US

## Abstract

**Background:**

Psychopathology in women after childbirth represents a significant risk factor for parenting and infant mental health. Regarding child development, these infants are at increased risk for developing unfavorable attachment strategies to their mothers and for subsequent behavioral, emotional and cognitive impairments throughout childhood. To date, the specific efficacy of an early attachment-based parenting group intervention under standard clinical outpatient conditions, and the moderators and mediators that promote attachment security in infants of mentally ill mothers, have been poorly evaluated.

**Methods/Design:**

This randomized controlled clinical trial tests whether promoting attachment security in infancy with the Circle of Security (COS) Intervention will result in a higher rate of securely attached children compared to treatment as usual (TAU). Furthermore, we will determine whether the distributions of securely attached children are moderated or mediated by variations in maternal sensitivity, mentalizing, attachment representations, and psychopathology obtained at baseline and at follow-up. We plan to recruit 80 mother-infant dyads when infants are aged 4-9 months with 40 dyads being randomized to each treatment arm. Infants and mothers will be reassessed when the children are 16-18 months of age. Methodological aspects of the study are systematic recruitment and randomization, explicit inclusion and exclusion criteria, research assessors and coders blinded to treatment allocation, advanced statistical analysis, manualized treatment protocols and assessments of treatment adherence and integrity.

**Discussion:**

The aim of this clinical trial is to determine whether there are specific effects of an attachment-based intervention that promotes attachment security in infants. Additionally, we anticipate being able to utilize data on maternal and child outcome measures to obtain preliminary indications about potential moderators of the intervention and inform hypotheses about which intervention may be most suitable when offered in a clinical psychiatric outpatient context.

**Trial registration:**

Current Controlled Trials ISRCTN88988596

## Background

Previous research has shown that infants whose mothers suffer from mental disorder are at increased risk for developmental delays, cognitive and functional impairments, physical symptoms and injuries, as well as behavioral and emotional problems in pre-school and school age [1-6]. Affective and mood disorders, anxiety disorders, posttraumatic stress disorders, obsessive-compulsive disorders, and psychosis with elevated incidences of comorbid diagnoses, varying in onset, course and prognosis, have a higher prevalence in mothers, both postpartum and during early child-age years [7-17]. In clinical populations, co-occurring personality disorders have to be considered as a crucial part of the global risk that mentally ill mothers experience [18-21].

The quality of parenting has been viewed as one key mediator of the relationship between maternal psychopathology and the outcome for infants and young children. Maternal psychological and vegetative symptoms, such as social withdrawal, anxiety, emotional lability, impulsivity, and severe exhaustion or weariness, have been found to impinge on the interaction and the development of the relationship between mother and infant. Often, such mothers are emotionally, cognitively and/or behaviorally inhibited or impaired in their ability to recognize and react with appropriate “sensitivity” and “responsiveness” [22] to their children’s needs. In regard to the infants of these mothers, higher incidences of behaviors, such as persistent crying, motoric restlessness, averted gaze or head position, physical neediness and a lack of expression of delight in the presence of their mothers, have been observed [23-25]. These behaviors increase the mother’s experience of stress, which further contributes to the maintenance of maladaptive interactional behavioral patterns [26-32]. Without improvement in these negative interaction cycles, for example, through early interventions, there is a greater likelihood for infants to develop an insecure-avoidant or an insecure-ambivalent or disorganized attachment strategy during the first year of life [33,34]. According to Ainsworth et al. [22], in the Strange Situation, insecure-avoidant (Category A) infants do not actively seek proximity and physical contact to their mothers after separation, instead favoring exploration. Insecure-ambivalent (Category C) infants, on the other hand, tend to cling to their caregivers and are characterized by frantic appeals to establish and maintain close proximity to them. At the same time, they show anger and sulky or aggressive behavior towards their mothers. These children are difficult to comfort after separation, that is, they maximize their attachment neediness [22]. Secure infants (Category B) reach out to their mothers at times of separation distress and calm down easily when comforted so that they can resume play or exploration. If the context of care is additionally characterized by either fear-inducing or frightening parenting practices (e.g., abuse, neglect, aggressive behaviors) or by fearful behaviors on the part of the mother (e.g., signs of anxiety or avoidance, dissociation, etc.), the child is more likely to develop disorganized or disoriented behavioral patterns (Category D) [35,36]. These include contradictory behavioral tendencies, such as fearful/anxious vacillation between exploring and seeking closeness, temporally uncoordinated or slowed movements and occasional physical or mimic paralysis or “freezing” of the child toward the mother. A maternal mental illness is associated with the development of insecure organized (avoidant, ambivalent) and disorganized attachment styles in the child [37-41]. Social-emotional maladjustment and related mental health problems in the child are common consequences [42,43]. Furthermore, mothers with mental disorders themselves are frequently characterized as insecure (dismissing Category Ds or preoccupied Category E), or unresolved (Category U) rather than autonomous (Category F) when attachment representations of their own childhood experiences with attachment figures are assessed [44-47]. These mothers are troubled by the physical and affective states, needs and behaviors of their infants, which may correspond to “frightening and frightened” [48] and/or “disrupted” [49] parenting behaviors [50].

According to the model of transgenerational transmission [51], maternal attachment representations influence the child’s attachment more than maternal sensitivity does [52-54]. Further clinical research on this issue provides evidence that the maternal capacity for mentalization appears to be more crucial than the expressed sensitivity of mothers in the attachment relationship [55]. The mental abilities of the mother to perceive and recognize her own and the child’s wishes, motives, needs, thoughts and feelings within the context of the attachment relationship and to communicate, as well as to reciprocate these through active engagement, kinesthetic expressions, words and play, represent key competencies for the development of secure attachment, self-regulation and mental health in the child – even if the mother had experienced unprocessed adverse experiences during her own childhood [56]. Conversely, a diminished capacity to mentalize brought about, for example, by maternal psychopathology, increases the likelihood of the child developing an insecure or disorganized attachment style, unless this process can be changed by an attachment-based intervention.

Research on the treatment of women with postpartum depression has shown that treatment of only the mother (i.e., medication, individual psychotherapy) is not sufficient to buffer against the negative impact of psychopathology on the child’s cognitive and psychosocial development, as well as attachment [57-61]. Rather, there is a necessity to support mentally ill mothers in their specific needs in caring and relating to their infants. Attachment-based interventions rooted in empirical research on developmental psychopathology are, thus, promising approaches to address these multi-faceted treatment targets. However, to date, very few attachment-based interventions have been systematically tested in formal clinical settings using a randomized controlled trial (RCT) framework involving women with complex postpartum psychiatric disorders.

Attachment-based interventions are designed to promote maternal sensitivity, to change maternal mental representations, to promote attachment security in the child, and/or to support the family, examples include STEEP™ [62]; Wait, Watch, and Wonder [63]; Circle of Security [64]; Video Intervention To Promote Positive Parenting [65]. Each of these interventions has been conducted in different settings (home-based, institutional, individually, group-based), with various at-risk populations (i.e., low SES, depressed mothers, adolescent mothers, preterm infants), and at a wide range of dosages and intensities. Video-analysis is a typical technique implemented as part of these interventions in order to facilitate change. Meta-analytic evidence identifies short-term approaches (< 16 sessions), targeting maternal sensitivity as being the most effective, and sensitivity-focused interventions conducted with clinically referred samples (i.e., *DSM-III-R* depressed mothers), as being more effective than interventions with other groups [66]. Attachment security, in particular, has been found to be readily influenced by sensitivity-focused interventions [66]. Yet, it remains unknown how brief attachment-focused interventions designed for at-risk populations differentially impact mothers’ mentalizing, mental representations of attachment, beyond sensitivity and mental illness, and children’s level of attachment security.

The Circle of Security intervention is a brief, behavioral and insight-oriented therapeutic group approach for promoting attachment and autonomy in the parent–child relationship. It combines psycho-educational, cognitive-behavioral, and psychodynamic understanding and intervention techniques. Given that women with various and co-occurring mental illnesses after childbirth differ greatly in their requirements, openness and motivation for, and compliance with mother-infant treatment, the COS intervention can allow individualized, flexible and deepened therapeutic access to each mother-infant dyad, which is further supported by its group character. Therefore, within our clinical context, COS seems to be a promising intervention to detect and treat early difficulties in the development of secure attachment relationships.

The current RCT is designed to evaluate the efficacy of the COS intervention for mentally ill mothers with infants, for the first time in Germany and in a clinical context. The main research question is whether COS, in comparison with treatment as usual (TAU), increases attachment security and prevents the development of insecure and/or disorganized attachment by promoting maternal sensitivity and mentalizing measured after treatment at follow-up when the children are aged 16–18 months. We hypothesize that following treatment there will be a higher proportion of secure child–mother attachment in the COS arm compared to TAU. This paper describes the design of the trial, the implementation of the study protocol in a child and adolescent psychiatric outpatient unit, and the data analysis strategies.

## Methods and design

### Overview

The Circle of Security Study in Hamburg represents a clinically pragmatic form of prevention and intervention, performed in a RCT. The study is comparing two mother-infant treatment groups, assigned to COS or TAU as the control condition, in the outpatient unit at the Department of Child and Adolescent Psychiatry, Psychotherapy, and Psychosomatics at the University Medical Center of Hamburg. The recruitment, baseline and follow-up assessment of mother-infant dyads started in January 2010 and will end in April 2014. Participants were recruited from among those mothers visiting the outpatient unit who volunteered to take part, and after having given their informed consent, entered the baseline assessment for this study. Mothers with mental disorder and their infants attending the outpatient unit were referred to the unit from other medical/mental health services they had been accessing from different areas of Hamburg, an urban city with a population of approximately 1.8 million inhabitants.

### Aims and hypothesis

The primary aim of this study is to evaluate the efficacy of COS versus TAU in mothers with mental disorder and their infants in promoting attachment security in the child under standard clinical outpatient conditions. More specifically, the goal of the study is to determine whether COS intervention results in more securely attached infant–mother dyads than the control condition (TAU). The primary hypothesis is that the COS intervention will bring about a higher rate of securely attached children after treatment and at follow-up (when the children are aged 16–18 months) than TAU. Secondary hypotheses being tested by the study are that i) COS intervention will lead to an increase in sensitive behavior in mothers, ii) COS intervention will result in an increase in mothers’ ability to mentalize, and iii) COS intervention will improve mothers’ state of mind with regard to their own attachment, relative to TAU.

### Participants

All eligible mother-infant-dyads seeking mother-infant treatment were invited to participate in this study, and were entered into the baseline assessment after both parents having given their informed consent. The inclusion criterions were again verified, and the mother-infant dyads then randomly assigned to the treatment arms. Demographic information, such as socioeconomic status, as well as age, education, and treatment history of the mothers were assessed at baseline. All mother-infant dyads were German-speaking residents of the greater Hamburg area.

#### Eligibility criteria

The inclusion criteria are child age 4–9 months and mothers’ being a fluent speaker of German. The exclusion criteria are child autism and early retardation or a primary ICD-10/DSM-IV diagnosis of substance abuse, schizophrenia, intellectual impairments (IQ < 80), or suicidal ideation and/or recent suicide attempt in mothers. No other exclusions were made in order to ensure that a full and representative spectrum of mentally ill mothers with infants attending our outpatient unit for was included.

### Interventions

This trial aims to evaluate the efficacy of the COS group treatment protocol compared to TAU as the control condition. The COS group intervention consisted of 20 90-minute sessions delivered weekly on Tuesday afternoons, plus one additional session of 120 minutes with the fathers or close family members on one Saturday. The TAU condition was delivered at different levels of intensity, as needed by the mothers. Mothers assigned to either of the treatment arms were permitted to receive psychopharmacological medication as part of their adult psychiatric treatment to manage clinical symptoms, as well as individual psychotherapy and/or other psychosocial services.

#### Circle of Security (COS) intervention

The Circle of Security (COS) intervention [64,67] was designed to alter developmental pathways for at-risk parents and their children. In the current study, COS was only used with mothers, except for the single additional session with fathers/family members. It was conceptualized as a psychodynamically oriented, psychotherapeutic and community-based parenting program and is considered to be evidence-based in the USA. The COS is a treatment based on groups of six individuals. The group intervention focuses on the caregiver and his/her relational capacities in providing child–parent attachment security. The COS manual (unpublished) draws upon the work practiced in Early Head Start and Head Start programs in the USA and was developed by the originators [68] at the Marycliff Institute in Spokane, WA. The COS therapists of the current trial underwent an intensive 10-day training in COS assessment and treatment organization at the Marycliff Institute in preparation for this study.

Prior to the beginning of the group therapy, a thorough diagnostic assessment of the interactions between mother and infant in various situations was performed, during which attachment and explorative behaviors were activated. Additionally, an interview with regard to caring for the child (the “Circle of Security” Interview; [69]), as well as an attachment interview (Adult Attachment Interview, AAI; [70]) were conducted with the mother. With the “Circle of Security” model as a foundation for intervention, an assessment of the relationship between mother and child was prepared with the help of video recordings and both interviews. From this assessment, the maternal core difficulty (termed the “linchpin struggle”) and the preferred (unconscious) defense strategy against potential threatening or uncomfortable emotions (procedural) or cognitions (termed “shark music”) connected with the attachment and autonomy needs of the child, were deduced. An individual treatment plan was formulated for each mother-infant-dyad,. This treatment plan contained a sampling of video sequences, which were used throughout the therapeutic process in order to acquaint the mother with her “linchpin struggle” whilst taking the mother’s “core sensitivity” and “shark music” into account. The “core sensitivity” describes an integral aspect of the personality [71,72], which represents the emotional organization of relationships and the basis for defensive processes in the parent–child interaction. The need for relationships and the fear of the loss of these relationships or separation play central roles in this. Each mother’s idiosyncratic manifestation of this complex interplay was taken into consideration in the selection of the video sequences.

Every therapeutic group consisted of six women. Six groups were planned and realized for this trial. The infants did not participate in group sessions, and childcare was provided for them while the mothers were in sessions.

The treatment manual, which was designed for parents with children aged 0–5 years, combines psycho-educational and therapeutic modules (e.g., video analyses in the group), focusing on attachment, exploration, communication and affective experiences between parent (in this study, mother) and child, aiming to help the mothers to elaborate their strengths and struggles in order to create a haven of safety and a secure base for their children. Guided by the treatment manual, there is a shift from predominantly observing behavioral and affective cues of both mother and child to gaining more insight into maternal thoughts and feelings and their defensive nature (“shark music”) with regard to the attachment and autonomy needs of the children, with the goal of overcoming their impact on child-mother attachment formation and maternal sensitivity. This is intensified by showing video segments of the “linchpin struggle” of each mother-infant dyad.

The 90-minute-COS group sessions took place weekly. Two therapists facilitated each group. Treatment adherence and integrity in this study was assured by weekly supervision of the therapists with Bert Powell, one of the originators of the COS intervention.

#### *Treatment* a*s usual (TAU)*

As a control condition, TAU was provided to mothers with their infants within the child and adolescent psychiatric outpatient unit. TAU consisted of standard treatment, which may have involved case management or counseling for the mothers. The treatment was offered by two child and adolescent psychiatrists and psychotherapists with a theoretical blend of psychodynamic and behavioral training in therapeutic work with mentally disordered mothers and infants. The focus was on the mother, the child, and/or the mother–child relationship; fathers were sometimes included. Each treatment session typically lasted 50 minutes. The intensity and length of treatment depended on the specific needs of each mother–infant dyad, and treatment was terminated by the mothers. The exact number of treatment sessions was recorded. Mother–infant dyads with particular needs were referred for additional services (e.g., social support services, family midwives). Most of the mothers attending the outpatient unit had seen an adult psychiatrist to confirm their diagnosis and further treatment indications at least once, at the beginning of treatment. At the time of follow-up, treatment satisfaction will be assessed for all mothers in the COS and TAU groups.

### Assessment and outcomes measures

All measures of pre- and post-assessment are listed in Table 1.

**Table 1:** Assessments administered at baseline and follow-up throughout the trial

**Time point**	**SSP**	**MBQ-S**	**DIP**	**AAI**	**AAI-RF**	**PRFQ-1**	**SCID-I,-II**	**BDI**	**SCL-90**	**PSI**	**DERS**	**CBCL**	**FBB-E**
**Baseline**													
Child age 4–9 months	**-**	√	√	√	√	√	√	√	√	√	√	√	**-**
**Follow-up**													
Child age 16–18 months	√	√	√	√	√	√	**-**	√	√	√	√	√	√

#### Primary outcome

##### Child attachment

The primary outcome of the current study is child attachment security (vs. insecurity). In attachment research, the Strange Situation Procedure [SSP; 22] is the most widely used and well-validated experimental paradigm for assessing the quality of the child’s attachment to a parent in infancy. The ratings for attachment qualities will be done by two trained and reliable coders blind to treatment allocation. The child’s attachment behaviors will be rated for classification into secure-B, or into avoidant-A or ambivalent-C as insecure-organized attachment qualities, according to the standard criteria described by Ainsworth [22], and for classification into insecure-disorganized/disoriented-D attachment qualities, according to the Main and Solomon [35] coding system.

#### Secondary outcomes

**Mini-MBQS** - Maternal sensitivity. The revised mini-Maternal Behavior Q-Sort (mini-MBQS) for video coding [73], a 25-item observational coding instrument, is used for measuring the quality of maternal sensitive behavior. The q-set items relevant to attachment are mapped onto a prototypically sensitive mother [74]. During the mini-MBQS sorting process, coders systematically evaluate and sort each of the 25 q-set items into one of the five categories (i.e., “most like mom”, “like mom”, “unlike mom”, “neither like nor unlike mom, “least like mom”) respectively, based upon behavioral observations of videotaped interactions between mother and child. Correlation scores are calculated, varying from -1.0 (least sensitive or responsive) to 1.0 (prototypically sensitive or responsive) between the observer sorts and a criterion sort for the prototypically sensitive and responsive mother, which is provided by the developers of the instrument. In this trial, mini-MBQS data are derived from videotaped 5-minute free play and 5-minute book reading interactions at baseline and follow-up, each coded by two independent raters blind to treatment allocation.

**DIP** - The Disconnected and extremely Insensitive Parenting rating procedure [75] is used to identify extreme insensitivity and/or disconnection in the mother. The DIP captures those maternal behaviors that are most likely related to poor child attachment outcomes [75]. Disconnected behavior and extreme insensitivity are rated on a scale ranging from 1 to 9, indicating an incrementally increasing display and/or prolonged duration of such behaviors. For pre- and post-assessment, the DIP is administered for the same 5-minute-interaction episodes (free play, book reading) as the mini-MBQS by two independent raters who each have undergone DIP rating and reliability procedure training.

**AAI** - The Adult Attachment Interview and its respective scoring and classification system [70,76] represent the “gold standard” for classifying maternal state of mind with regard to attachment (as F, Ds, E, U). In the current trial, it is administered at baseline and follow-up. Mothers classified as unresolved (U) with regard to loss or trauma will also be assigned into a secondary best-fitting organized attachment category. The interviews will be transcribed verbatim and coded by two reliable raters who are blind to treatment allocation.

**RF -** The Reflective Functioning (RF) scale of the AAI [77] is utilized to measure maternal mentalizing, which is defined as the capacity of the mother to reflect upon her own self and close others in terms of intentional mental states. The RF allows for overall ratings of reflective functioning on a nine-point scale ranging from 1 (lacking RF) to 9 (exceptional RF), with two additional RF scores indexing the absence of mentalizing, that is, -1 (negative RF), 0 (lacking RF). RF-ratings will be performed on the AAI obtained at baseline and follow-up by two independent raters blind to treatment allocation.

**PRFQ-1 –** The Parental Reflective Functioning Questionnaire-1 [78] is used as an alternative instrument for assessing RF via self-report. The PRFQ-1 assesses the levels of parental mentalization up to the child’s age of 3 years. It consists of 39 items comprising three subscales prototypically describing high, low, and neither high nor low mentalizing in mothers and fathers. Scoring procedures precepts yield a total score on all three subscales. PRFQ-1 is administered at baseline and follow-up.

**SCID-I** – The German version of the Structured Clinical Interview [79] for DSM-IV [80] axis I disorders is used to verify clinical DSM-IV-Axis-I diagnoses. SCID-I interviews were conducted by trained researches at baseline.

**SCID-II** – The German version of the Structured Clinical Interview (SCID-II) for personality disorders is used to identify mothers with probable DSM-IV-Axis II personality disorders. SCID-II interviews were conducted by trained researchers at baseline.

**BDI** – The Beck Depression Inventory [81,82] is administered to screen for depressive symptoms in the mothers at baseline and follow-up. It consists of 21 items, scaled from zero to three, which are summed to a global score, indicating clinical significance of self-reported depressive symptoms.

**SCL-90-R** – The revised Symptom Checklist by Derogatis [83] (German version: [84]) is used to assess maternal psychopathological symptoms. The Global Severity Index, as a sum score of the SCL-90-R, is applied to determine maternal psychopathology at baseline and follow-up.

**PSI** – The Parenting Stress Index [85] (German: [86]) is a self-report measure consisting of 120 items for parents with children aged from 0–12 years, capturing child-related and parent-related stress domains. A total stress score is derived, indicating the severity of the subjective burden of parenting. A domain of life events allows for the recording of positive and negative life events. The PSI is measured at baseline and follow-up.

**DERS –** The Difficulties in Emotion Regulation Scale [87] is a 36-item self-report on clinically relevant emotion regulation difficulties, especially with regard to negative emotions. Items are scored on a 5-point Likert scale ranging from 1 (almost never) to 5 (almost always). The authors have confirmed a six-factor structure of emotion regulation, upon which the DERS is based; and adequate internal consistency (Cronbach’s α > .80) of these subscales has been established [87]. For the purpose of this trial, the DERS was translated into German. First, the scale was translated from English to German independently by two translators who then discussed their translations and combined them to form a draft of the questionnaire used in the present study. This draft was back-translated into English by a professional translator. The back-translated version was found to be consistent with the original DERS version. The DERS is applied at baseline and follow-up.

**CBCL** – The Child Behavior Checklist1½**-**5 [88] (German version) is a questionnaire for parents of children aged 1.5-5 years, which is used to assess child behavioral and emotional problems. The items are related to seven problem scales, loading on externalizing, internalizing, and a total score. In the current trial, the CBCL will be administered at follow-up.

**FBB-E** – Fragebogen zur Beurteilung der Behandlung – Eltern [89] (“A Questionnaire to Evaluate Treatment Satisfaction – Parent Version”) is a well-known German language questionnaire for evaluation and quality assurance of the therapeutic treatment of children, adolescents and their families. The FBB-E assesses how positively or negatively parents evaluate the course and outcome, as well as their personal satisfaction regarding the psychotherapeutic treatment of their children, independent of treatment modality. The FBB-E will be applied at follow-up.

### Randomization

#### Recruitment and baseline procedures

**Figure 1:**
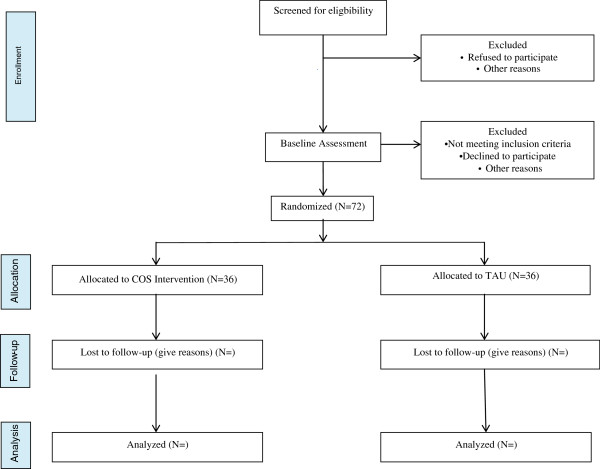
Consort diagram describing flow of patients through study.

The flow of participants from recruitment through to the end of the study is shown in Figure 1.

Identification and initial screening of potential participants were performed by the principle investigator of the study, an experienced clinician in the outpatient unit. Once eligible mother-infant dyads were identified, they were informed about the trial and invited to participate. Both custodial parents had to give their informed consent for the baseline assessment, which was performed by trained research staff over several sessions. After completion, the researcher confirmed whether each mother met diagnostic and additional inclusion criteria. If they did, they were randomly assigned to one of the two treatment arms (COS or TAU). Once consent to treatment and post-assessment of both parents had been obtained, a trial ID was assigned. Depending on the natural flow of patients a COS group began once six mother-infant cases had been recruited and assigned. TAU started immediately after random allocation. Follow-up assessment (child age 16–18 months) is continuing.

#### Randomization procedures and methods to minimize bias

Eligible patients were randomly assigned to the COS intervention or TAU in a ratio of 1:1. An electronic randomization list was generated using Randlist 1.2 as a random generator. Unique randomization numbers were generated to ensure that the study arm assignment was unbiased. Randomization was stratified for two infant age groups (4–6 months vs. 7–9 months) and separate random lists were created for each age group. Block sizes of six to eight were used for the randomization of the treatment arms. The randomization list was generated by the randomization representative and was stored in a password-protected electronic document accessible only by the randomization representative. Each random number was stored in a concealed randomization envelope. After a patient’s eligibility for the study was determined, the next available randomization number was assigned to the patient, in ascending order. To minimize bias that could have arisen from knowledge of treatment allocation, all outcome assessors were blind to treatment allocation and a random sample of outcome measures was re-rated by independent assessors.

### Statistical methods

#### Sample size and power

The primary outcome for treatment efficacy is the rating of quality of child attachment at follow-up at children’s age 16–18 months (COS vs. TAU) by an objective rater blind to treatment allocation. Differences in distributions of the quality of child attachment (secure vs. insecure-avoidant, -resistant, -disorganized) between both treatment modalities are calculated by a chi-square test of significant statistical independence. A power analysis for comparing COS and TAU with a power of 80% and the probability of making a Type I (α) error of 5%, with a medium effect (*W* = 0.3) suggested a recruited sample size of N = 80.

#### Statistical analysis plan

All available variables will be used without missing outcomes being factored in. The analyses are based upon the assumption of ignorable drop-outs. Characteristics of the treatment groups were assessed at baseline.

### Primary outcome

For the primary outcome, that is, child attachment security (vs. insecurity) as a categorical variable, differences of the proportions of child attachment security between TAU and COS treatment arms will be estimated by a chi-square test of statistical significance.

### Secondary outcomes

a. To compare differences between COS and TAU on maternal sensitivity (MBQS, DIP) as continuous secondary outcome variables, a 2x2 analysis of covariance (ANCOVA), with repeated measurement (t0-t1) on one first factor, will be used. In these analyses, treatment modality will be treated as the second factor, maternal sensitivity as the dependent variable, and maternal psychopathology (BDI, SCL-90-R) and maternal parenting stress (PSI) as co-variables.

b. To compare differences between COS and TAU on maternal mentalization (RF, PRFQ-1) as continuous secondary outcome variables, a 2×2 analysis of covariance (ANCOVA), with repeated measurement (t0-t1) on one first factor, will be used. In these analyses, treatment modality is to be treated as the second factor, maternal mentalization as dependent variable, and again, maternal psychopathology (BDI, SCL-90-R) and maternal parenting stress (PSI) as co-variables.

c. Furthermore, in a multiple linear regression, the differences in maternal sensitivity and mentalization will be used to predict psychopathology and maternal stress (with treatment modality, differences in psychopathology, and differences in maternal distress as independent variables).

d. Before analyzing the proportions of mothers whose attachment representations change during the treatment and follow-up period in both treatment arms, we will separate those mothers with a positive shift in attachment representations [i.e., insecure (-dismissing, preoccupied, unresolved) to secure) from the mothers without such a shift or with a negative one ((i.e., secure to any of the others)]. For binary responses, a binary logistic regression will be used to compare proportions of mothers whose attachment representations (positive shift vs. no/negative shift) have been altered as a criterion variable, and treatment group (COS vs. TAU), differences of maternal psychopathology (BDI, SCL-90) as well as parenting stress (PSI) prior and after treatment as predictor variables.

e. To examine associations between changes of psychopathology (BDI, SCL-90) and changes in parenting stress (PSI) between pre- and post-assessment, a Pearson correlation analysis with the difference scores will be conducted.

f. Child behavior and emotional problems (CBCL11/2) at follow-up will be analyzed with respect to mean scores in either arm of the trial, using multiple linear regression analyses. Again, maternal psychopathology (BDI, SCL-90) and maternal parenting stress (PSI) will be treated as predictor variables, in addition to treatment modality.

g. Further multiple regression analyses will be conducted, with mean changes in maternal sensitivity (MBQS, DIP), mentalization (RF, PRFQ-1), and maternal attachment representations (positive shift, no/negative shift) before and after treatment, as predictor variables.

h. Binary logistic regression models will be used to compare proportions of child attachment security (vs. insecurity) at follow-up as criterion variables, using treatment modality, maternal sensitivity, maternal self-reflection (RF), maternal psychopathology (BDI, SCL-90), and maternal parenting stress (PSI) as predictor variables.

## Ethics

The trial received ethical approval from the local ethics committee of the Medical Board of Hamburg, Hamburg, Germany on 24 August 2009 – reference number PV3269.

## Discussion

This RCT is notable in that, through a high-quality design, it evaluates an attachment-based parenting program, namely the COS intervention, in terms of its potential to promote early attachment security in infants of women with mental disorders, who are at increased risk for unfavorable attachment and developmental outcomes. Comparing the attachment outcomes in infants of mothers with psychopathology who received COS or TAU will determine the relative benefit to the child associated with each of the interventions. Furthermore, by collecting data concerning the mother’s psychopathology, parenting behaviors (i.e., sensitivity), mentalizing and attachment representations, both at baseline (at child’s age 4–9 months) and at follow-up (at child’s age 16–18 months), we will be able to test factors that might influence treatment responses on the transmission model.

This is the first trial of COS within a child and adolescent psychiatric outpatient unit. Therefore, this trial will provide an opportunity to examine gains resulting from COS as a clinical preventive and therapeutic tool for infants and their mothers with mental illness.

### Limitations

The circumstances of this clinical trial necessitated some scientific compromises. One limitation of this trial is that the person who oversaw the implementation of the COS intervention is also the principle investigator. Thus, personal factors (allegiance effects) may limit the generalizability of our findings. In view of the fact that the study sample comprises clinically referred mothers with mental disorders and their infants, it is ethically unacceptable to use a no-treatment control group. Therefore, in the best interest of our patients we have used TAU as the control intervention to which patients are randomized. According to the natural flow of patients attending the outpatient unit, eligible women with infants participated voluntarily, and, having given their informed consent, were randomly assigned to COS or TAU. Thus, our clinical sample varied in terms of psychopathology. There may be a sampling bias, insofar as the mothers with infants attending the outpatient unit might represent a more severely mentally disordered sample choosing to participate in this trial. This may limit the generalizability of the findings, due to sampling bias and a lack of true representativeness. Hence, replication studies or a multi-site trial are necessary. A sample size of N = 80 was determined before any data were collected, based on the assumption of medium effect size and power. For practical reasons, we defined a sample size of N = 72, such that 36 mother-infant dyads were randomly assigned to COS (six COS therapeutic groups) and 36 mother-infant dyads to TAU. Further long-term follow-up measures might be useful to capture positive changes of the intervention that may be attributable to a sleeper-effect [66] and apparent only at later follow-up stages.

## Competing interests

The authors declare that they have no competing interests.

## Authors’ contributions

BR and BP designed the trial. BR wrote the grant application together with GR and AL. BR, PF and TN designed the data analysis strategy, AL and CM contributed to calculating statistical power for the primary outcome variable and writing the statistical and data analytical plan together with BR, PF, and TN. All authors read and approved the final manuscript.

## Pre-publication history

The pre-publication history for this paper can be accessed here:

http://www.biomedcentral.com/1471-244X/14/24/prepub
